# Connaissances, attitudes et pratiques des femmes en âge de procréer du District de Santé de la Mifi sur la prévention du cancer du col de l’utérus, Cameroun

**DOI:** 10.11604/pamj.2018.31.172.16320

**Published:** 2018-11-12

**Authors:** Rama Djouedjon Dakenyo, Bruno Kenfack, Noel Vogue, Eva Fomo Tsakoue, Maurice Ela Ebode, Samuel Nambile Cumber

**Affiliations:** 1 Faculté de Médecine et des Sciences Pharmaceutiques, Université de Dschang, Cameroun; 2 Délégation Régionale de la Santé Publique du Centre, Yaoundé, Cameroun; 3 Faculty of Health Sciences, University of the Free State, Bloemfontein, South Africa; 4 Section for Epidemiology and Social Medicine, Department of Public Health, Institute of Medicine (EPSO), The Sahlgrenska Academy at University of Gothenburg, Gothenburg, Sweden

**Keywords:** Connaissance, attitude et pratique, prévention, cancer du col de l´utérus, knowledge, attitude and practice, prevention, cervical cancer

## Abstract

**Introduction:**

Décrire les connaissances, les attitudes et les pratiques des femmes en âge de procréer sur la prévention du cancer du col de l'utérus.

**Méthodes:**

Il s'est agi d'une étude transversale descriptive menée chez les femmes en âges de procréer. Les données ont été collectées face à face à l'aide d'un questionnaire. L'analyse a été faite par épi info version 7.1.3.3 avec un intervalle de confiance de 95%.

**Résultats:**

Il ressort de notre étude que 78,11%(464/594) de femmes connaissaient l'existence du cancer du col de l'utérus. Mais 58,59% et 60,27% de femmes ne connaissaient aucun facteur de risque et moyen de prévention respectivement. Seulement 7% connaissent l'existence d'un vaccin. Concernant l'attitude, 31,31% (186/594) avaient déjà désiré les informations sur la prévention du cancer du col de l'utérus, 26,94% (160/594) ont eu l'initiative de faire le test de dépistage et seulement 7,41% (44/594) ont fait le dépistage. Les difficultés évoquées étaient principalement le manque d'information 69,82% et le moyen de sensibilisation le plus fréquemment cité était les medias 75,9%.

**Conclusion:**

Malgré le bon niveau de connaissance sur l'existence du cancer du col de l'utérus, le niveau d'information sur les facteurs de risque et les moyens de prévention restent faibles. Le programme de sensibilisation devait être axé sur les facteurs de risque, les moyens de prévention et l'organisation des campagnes de dépistage. Ces informations peuvent permettre d'améliorer la planification des interventions de prévention.

## Abstract (en)

**Introduction:**

This study aims to describe knowledge, attitudes and practices of women of childbearing age with regards to cervical cancer preventive measures.

**Methods:**

We conducted a cross-sectional descriptive study among women of childbearing age. Data were collected using a face to face questionnaire and were analyzed using Epi Info 7.1.3.3 software calculating a confidence interval of 95%.

**Results:**

Our study highligts that 78.11%(464/594) of women were aware of the existence of cervical cancer. However 58.59% and 60.27% of women were not aware of any risk factor and preventive measure respectively. Only 7% of these women were aware of the existence of vaccine. With regard to their attitude, 31.31% (186/594) of women had already wished for information on cervical cancer preventive measures, 26.94% (160/594) had taken the initiative to undergo screening test and only 7.41% (44/594) had undergone screening test. Difficulty most commonly mentioned was the lack of information (69.82%) while the most commonly cited awareness-raising tools were the media (75.9%).

**Conclusion:**

Despite the good knowledge on the existence of cervical cancer, the level of information about risk factors and preventive measures remains weak. Awareness program should be focused on risk factors, preventive measures and on the implementation of screening campaigns. These informations can be used to improve planning approach to prevention.

## Introduction

Les cancers gynécologiques constituent les cancers les plus fréquents et les plus graves parmi la population féminine mondiale [1]. Le cancer du col de l'utérus par sa fréquence, se place au deuxième rang des cancers féminins dans le monde après le cancer du sein. C'est la quatrième cause la plus fréquente de décès par cancer (266 000 décès en 2012) chez les femmes dans le monde [2, 3], On note 528 000 nouveaux cas de cancer du col de l'utérus chaque année. En Afrique, on observe des taux extrêmement élevés de cancer du col (plus de 50/100.000 femmes) [4]. Cette forte prévalence en Afrique est liée au niveau de développement dont certainement du niveau de connaissance des populations sur les moyens de prévention du cancer du col de l’utérus. Au Cameroun en 2012, le registre du cancer de Yaoundé avait estimé l’incidence ajustée selon l’âge à 107 nouveaux cas pour 100.000 habitants, le cancer du col de l’utérus occupait le deuxième rang (13,8%) après celui du sein (18,5%) [5]. La population à risque de développer le Cancer du Col de l´Utérus (CCU), au Cameroun est de 6,74 millions de femmes âgées de 15 ans et plus. Selon les estimations actuelles, chaque année 1993 femmes sont diagnostiquées avec le cancer du col de l´utérus et 1.120 en meurent [6]. Les facteurs de risques et les moyens de prévention sont connus, leur maitrise par la population contribuerait à diminuer sa fréquence. En France les taux d'incidence et de mortalité par cancer du col de l'utérus sont en constante diminution [7] à cause de la diffusion à large échelle du dépistage précoce dans les pays développés. La stratégie actuelle de l'OMS recommande quand c'est possible de débuter le dépistage par le test HPV et continuer par les inspections visuelle à l'acide acétique ou au lugol avant de poursuivre par les frottis et biopsie [8]. Nous nous sommes proposés de décrire et d'analyser les connaissances, les attitudes et les pratiques des femmes en âge de procréer sur la prévention du cancer du col de l'utérus.

## Méthodes

Il s'est agi d'une étude transversale descriptive à base communautaire menées chez les femmes en âges de procréer (FAP) dans le district de santé de la Mifi.

$${\text{z}}^{2}{\text{P(1-P)/d}}^{2}$$

La formule statistique sus mentionnée [9] a été utilisée pour obtenir la taille minimale d'échantillon et 27,9% la prévalence des connaissances des femmes au dépistage du cancer du col de l'utérus d'une étude antérieure [10]. La taille minimale de 309 a été déterminée, cette taille a été réajustée en ajoutant 10% (taux de non réponse) à 340. En tenant compte d'un l'effet grappe de 2, nous avons obtenu notre taille d'échantillon de 680 FAP. Nous avons mené l'étude dans 12 Aires de Santé sélectionnées de façons aléatoire. Etait éligible pour l'étude les FAP (15-49 ans) des Aires de Santé sélectionnées et ont été inclus celles ayant consentis à participer à l'étude. Après avoir soumis le protocole au Comité National d'Éthique, le questionnaire a été prétexté. Les enquêteurs ont été recrutés et ont été formés. L'enquête s'est faite porte à porte et le questionnaire a été administré face à face. Nous avons collecté les informations sur les caractéristiques sociodémographiques, les connaissances, les attitudes et les pratiques sur la prévention du cancer du CCU (Cancer du Col de l´Utérus). Apres la collecte, les questionnaires ont été dépouillés et codés, le masque de saisi des données a été élaboré par EPI INFO version 7.3.1.1 ensuite les données ont été saisies puis analysées, le test statistique KHI2 a été utilisé pour tester les associations, le seuil de significativité a été fixé à 5% (p<0,05).

## Résultats

Le taux de participation était de 87,35% (594/680). L'âge moyen était de 29,69 ± 8,87 ans et le mode était 30 ans. Les répondantes avaient en majorité un niveau d'étude secondaire 61,28% et étaient constituées des célibataires et des mariées. La moitié des participantes venaient de la zone urbaine 58,92% (350/594) (Tableau 1). Concernant les connaissances, 78,11% (464/594) de femmes qui connaissaient l'existence du Cancer du Col de l´Utérus (CCU), les sources d'informations les plus cités étaient la formation sanitaire 61,42% (285/464) et les medias 46,12 (214/464). Sur les 594 répondantes 246 (41,41%) connaissaient au moins un facteur de risque, parmi elles, 44,72% (110/246) et 41,46% (102/246) ont identifié les IST et la multiplicité des partenaires respectivement. Cependant, seulement 7,72% (19/246) d'elles identifiaient le HPV comme principal facteur de risque du cancer du col de l'utérus. Sur les répondantes, 39,73% (236/594) de femmes connaissaient au moins un moyen de prévention du cancer du col de l'utérus, 69,91% (165/236) ont identifié l'examen de dépistage et seulement 7,63% ont cité le vaccin (18/236) (Tableau 2).

**Tableau 1:** Données sociodémographiques des participantes

Age	Fréquence absolue	Fréquence relative(%)
15-24 ans	192	32.32
25-34 ans	220	37.04
35-44 ans	120	20.2
45-49 ans	62	10.44
**Zone de résidence**		
Urbaines	350	58.92
Semi-urbaines	161	27.11
Rurales	83	13.97
**Niveau scolaire**		
Non renseigné	13	2.19
Non scolarise	20	3.37
Primaire	87	14.64
Secondaire	364	61.28
Supérieure	110	18.52
**Statut marital**		
Célibataires	283	47.64
Mariées	292	49.16
divorcées	15	2.53
veuve	4	0.67
**Parité**		
Nullipare	116	19.53
Primipare	142	23.90
multipare	336	56.57

**Tableau 2:** Connaissance sur le cancer du col de l’utérus

Le cancer du col utérin existe-t-il ? (N : 594)	Fréquence absolue	Fréquence relative (%)
Oui	464	78,11
Non	130	21,89
**Sources d’information N : 464**		
Média	214	46,12
Formation sanitaire	285	61,42
École	58	12,5
Internet	46	9,91
Culture personnelle	44	9,48
**connaissez-vous au moins 1 les facteurs de risques ? N : 594**	**Fréquence absolue**	**Fréquence relative (%)**
Oui	246	41,41
Non	348	58,59
Facteurs de risque cités N : 246		
HPV	19	7,72
Multiplicité des partenaires	102	41,46
Préciosité des rapports	38	15,45
Tabac	28	11,38
IST	110	44,72
Ménopause	22	8,94
Génétique	16	6,5
Vieillissement	14	5,69
**Existe-t-il les moyens de prévention du cancer du col utérin ? N : 594**	**Fréquence absolue**	**Fréquence relative (%)**
Oui	236	39,73
Non	358	60,27
**Moyens de prévention cités N : 236**		
Vaccin	18	7,63
Préservatifs	38	16,10
Examen de dépistage	165	69,91
Être fidèle à un seul partenaire	100	42,37
Éviter de fumer	32	13,56
Abstinence	44	18,64

Concernant l'attitude des femmes à la prévention du cancer du col de l'utérus, 31,31% (186/594) de femmes ont affirmé avoir déjà désiré les informations sur la prévention du cancer du col de l'utérus. Cependant, Il ressort que 26,94% (160/594) avaient déjà eu l'intention de faire le test de dépistage du cancer du col de l'utérus, 75% (120/160) ont été conseillées par un personnel de santé et seulement 16% (26/160) ont eu l'initiative personnelle (Tableau 3). Sur 594 répondantes, 44 ont affirmé avoir déjà fait le test de dépistage du cancer du col de l'utérus, soit une proportion de 7,41%. Parmi toutes les femmes enquêtées 96,13% (571/594) ont désiré obtenir plus d'information sur le cancer du col de l'utérus au moment de l'enquête. Les difficultés évoquées par les femmes étaient principalement le manque d'information 69,86% (415/594), ensuite la négligence 19,53%. Parmi nos répondantes, 448 ont suggéré les moyens de sensibilisation. Le moyen le plus fréquemment cité était les medias 75,9% (340/448) (Tableau 4). Ces résultats ont montré que plus de la moitié des femmes dans les différentes zone connaissait que le cancer du col de l'utérus existe. On note une association significative entre les connaissances et le niveau d'études ainsi que la zone de résidence. Les femmes ayant un niveau d'étude supérieure 97,27% (107/110) avaient de bonnes connaissances sur l'existence du Cancer du Col de l´Utérus. 86,75% (72/83) de femmes en zone rurale et 74,71% (65/87) des femmes ayant un niveau d'étude primaire ne connaissaient aucun facteur de risque du Cancer du Col de l´Utérus respectivement. On note une association significative entre la connaissance des facteurs de risque et le niveau d'étude ainsi que la zone de résidence. Parallèlement, concernant les connaissances sur les moyens de préventions, On note une association significative entre les connaissances et la zone de résidence ainsi que le niveau d'étude (Tableau 5).

**Tableau 3:** Attitudes sur la prévention du cancer du col de l’utérus

Avez-vous déjà désiré les informations sur la prévention du cancer du col de l’utérus? N : 594	Fréquence absolue	Fréquence relative (%)
Oui	186	31,31
Non	408	68,69
**Avez-vous déjà eu l’initiative de faire le test de dépistage du cancer du col de l’utérus ? N : 594**		
Oui	160	26,94
Non	434	73,06
**Si oui qu’est-ce qui vous a motiver à prendre cette initiative ? N : 160**	**Fréquence absolue**	**Fréquence relative (%)**
Personnel de santé	120	75
Ami	18	11,25
Initiative personnelle	26	16,25
Partenaire	10	6
Famille	8	5
**désirez-vous obtenir les informations sur le cancer du col de l’utérus ? N : 594**	**Fréquence absolue**	**Fréquence relative (%)**
Oui	571	96,13
Non	23	3,87

**Tableau 4:** Problème rencontrés par les femmes et proposition des moyens de sensibilisation

Problèmes rencontrés N : 594	Fréquence absolue	Fréquence relative (%)
Pas accès aux services de prévention	114	19,2
J’ai peur de connaitre mon statut	9	1,45
Manque de temps	58	9,82
Manque d’argent	52	9,45
Négligence	107	19,53
Manque d’information	415	69,86
**Moyens de sensibilisation sollicité (N : 448)**		
Les medias	340	75,9
Messages téléphoniques	56	12,5
Porte à porte	29	6,47
Affiche	68	15,18
Formation sanitaire	108	20,11
Causeries éducatives	62	13,83

**Tableau 5:** Influence des caractéristiques sociodémographiques sur les connaissances sur la prévention du cancer du col de l’utérus.

Caractéristiques socio démographiques	Connaissance sur les facteurs de risque du CCU			Connaissance sur l’existence du CCU			Connaissance sur les moyens de prévention du CCU		
	Oui	Non	X^2^ P	Oui	Non	X^2 ^P	Oui	Non	X^2 ^P
**Age**			0.55 0.45			0.32 0.5696			0,8 0,36
15-24 ans	144	48		74	118		79	144	
25-49 ans	320	82		172	230		157	244	
**Zone de résidence**									
Urbaine	292	58	13.71 0.001	148	202	17.81 0.0001	134	216	14,22 0,0008
semi urbaine	123	38		87	74		85	76	
rurale	49	34		11	72		17	66	
**Niveau d’étude**									
Primaire	60	27	18.09 0.0004	21	65	19.89 0.0002	18	70	19,67 0,0002
Secondaire	274	90		145	219		144	220	
Supérieure	107	3		72	38		68	42	
**Parité**									
Nullipares	85	31	0.82 0.6634	52	64	0.40 0.8183	48	68	1,47 0,48
Primipares	110	32		56	86		65	77	
Multipares	269	67		138	198		123	213	

## Discussion

L'étude que nous avons menée avait pour objectif de décrire les connaissances, les attitudes et les pratiques des femmes en âge de procréer sur la prévention du cancer du col de l'utérus dans le District de Santé de la Mifi. Le cancer du col de l'utérus est une pathologie connue par 78,11% de femmes dans notre étude , c'est pratiquement la même proportion dans les recherches de Mbongo au Congo concernant les connaissances, attitude et pratique sur le dépistage du cancer du col où 78,6% de femmes connaissaient l'existence du cancer du col de l'utérus [11]. Cette similitude peut s'expliquer par le fait que les caractéristiques socio- démographiques étaient presque les mêmes que celle de notre étude. Dans cette étude la plus part des femmes étaient multipares et l'âge moyen était 32,8 ans. La première source d'information sur le cancer du col dans notre étude a été la formation sanitaire 61,42% (285/464), contrairement à l'étude de mbongo au Congo où la première source d'information des femmes sur le cancer du col de l'utérus était les médias, cela peut se justifier par le fait que au Congo un projet de lutte contre le cancer du col de l'utérus par les médias a existé [11].

Dans notre étude, plus de la moitié des femmes ont affirmé ne connaitre aucun facteur de risque du cancer du col de l'utérus et 74,71% (65/87) des femmes ayant un niveau d'étude primaire ne connaissaient aucun facteur de risque contrairement à 65,45% (72/110) de femme ayant un niveau d'étude supérieure qui connaissaient au moins un facteur de risque du cancer du col de l'utérus. Nous avons trouvé qu'il existe une association significative entre les connaissances des facteurs de risque et la zone de résidence (Khi2 :17,81 et P: 0,0001). Cette association existe aussi avec le niveau d'étude. Cette situation peut s'expliquer par le fait que les femmes ayant un niveau d'étude supérieure et les femmes vivant en zone urbaine ont un niveau socioéducatif élevé et sont beaucoup exposées au service de santé et aux différents médias [12]. Parmi les femmes connaissant au moins un facteur de risque du cancer du col, 7,72% ont cité le HPV, ce résultat est presque similaire à celui des recherches de Assoumou au Gabon où 8,8% de femmes connaissaient le HPV [10]. Contrairement dans une étude faite en Australie où la vaccination Nationale au HPV et le programme d'information au HPV étaient disponibles, cette étude a montré que 88,9% de femmes avaient des connaissances sur le HPV [13]. La prévention primaire du Cancer du Col de l´Utérus par la vaccination a débuté dans notre pays et il devient intéressent déjà de sensibiliser la population en leur faisant savoir que le premier facteur de risque du Cancer du Col de l´Utérus est le HPV. Cette information est nécessaire pour la réussite d'une campagne de vaccination.

Les attitudes des femmes en matière de prévention du cancer du col étaient inadaptées dans l'ensemble. Seulement 31,31 % de femmes avaient déjà désiré les informations sur la prévention du cancer du col de l'utérus et 26,94% de femmes ont affirmé avoir déjà eu l'initiative de faire le dépistage du CCU, la principale motivation était le personnel de santé (75%). Dans une étude faite au Gabon, on a noté que 27,9% [10] de femmes avaient des informations sur la prévention du cancer du col et la principale motivation de faire le test de dépistage était le médecin (68,3%). Au Nigeria Obi *et al.* ont rapporté les mêmes résultats. Ceci montre une grande importance du personnel de santé dans la sensibilisation concernant la prévention du CCU. Dans notre milieu le nombre de médecin reste faible, l'unité de counseling / dépistage devrait être délégué au personnel infirmier spécialement formé pour la circonstance. On a noté une association entre le niveau le désir d'information sur la prévention du CCU, le niveau d'étude (Khi2:24,36, P: 0,00) et la zone de résidence (Khi2:4,94, P:0,08). Cette association était significative avec le niveau d'étude, le niveau d'instruction peut expliquer ces faibles attitudes, bien que nous n'ayons pas trouvé dans la littérature des données similaires. Cependant en Tunisie dans les recherches de Bouslah la situation n'était pas meilleure, le recours au dépistage du cancer du col de l'utérus était de 22,1%. [1] et, la pratique du dépistage était fortement corrélée à sa connaissance, à la participation aux séances d'éducation pour la santé et au niveau d'étude. Suite à la participation à ces séances, 98, 5% des femmes devenaient motivées à se faire dépister. 96,13% de femmes ont désiré obtenir plus d'information sur le cancer du col de l'utérus ceci peut s'expliquer par le fait que plusieurs femmes sont en quête d'information concernant le cancer du col de l'utérus.

Seulement 7,41% de femmes avaient déjà fait le test de dépistage, durant la période d'enquête. Plusieurs études ont montré une faible connaissance du HPV dans les pays en développement par rapport aux pays développés. Cette divergence nécessite le besoin d'informer la population sur le dépistage du cancer du col car la difficulté majeure citée par les femmes dans notre étude était le manque d'information à une proportion de 69,86% (415/594). Cela montre les difficultés pour les pouvoirs publics à assurer une couverture optimale de la population au dépistage et à responsabiliser les femmes pour une pratique régulière. Il est indispensable que dans l'organisation du dépistage qu'il y ait des campagnes d'information. Comme biais de l'étude nous pouvons avoir des biais d'information qui révèle du fait que certaines femmes peuvent avoir menti sur des questions.

## Conclusion

Le cancer du col de l'utérus est un problème majeur de santé publique au Cameroun. La plupart des femmes ont une connaissance sur son existence mais leur connaissance sur les facteurs de risque et les moyens de prévention est faible. Leurs attitudes et pratique sont également faibles. Le programme de sensibilisation sur le CCU, devait être axé sur les facteurs de risque, les moyens de prévention et l'organisation des campagnes de dépistage. Ces informations peuvent permettre d'améliorer la planification et l'élaboration des interventions de prévention.

### Etat des connaissances actuelles sur le sujet

- Le niveau d'information des femmes sur le cancer du col de l'utérus est bas;
- Les connaissances sur les causes, les facteurs de risque sont faibles;
- La pratique du test de dépistage est insuffisante.

### Contribution de notre étude à la connaissance

- Cette étude a permis de connaitre des facteurs qui influencent les connaissances sur le cancer du col dans les zones urbaines, semi urbaines et rurales;
- Cette étude a permis de connaitre les problèmes rencontrés par les femmes qui limitent leur connaissances sur le cancer du col;
- Cette étude a permis connaitre les moyens de sensibilisation sollicités par les femmes pour améliorer connaissances sur le cancer du col de l'utérus.

## Conflits d’intérêts

Les auteurs ne déclarent aucun conflit d'intérêts.

## Remerciements

Notre profonde gratitude va aux participants pour leur contribution.
